# Supplementary material for: Role of the Anaphase-Promoting Complex Activator Cdh1 in the Virulence of Cryptococcus neoformans
Source: J Fungi (Basel). 2024 Dec 23;10(12):891. doi: 10.3390/jof10120891 (PMC11678062; doi:10.3390/jof10120891)
Supplement: Supplementary file 1 [file jof-10-00891-s001.zip › jof-3270904-supplementary.pdf]

Supplementery tables

Table S1. Strains used in this study.

Strains	Genotype	Source/reference
E. coli		
DH5α	cloning strain	
C. neoformans		
H99	*MATα*	Perfect et al., 1993
	*MAT***a**	Nielsen K et al., 2003
KN99**a**		Nielsen et al., 2003
TBL508	*MATα cdh1Δ::NEO*	In this study
TBL509	*MAT***a** *cdh1Δ::NEO*	In this study
TBL512	*MATα cdh1Δ:: NEO CDH1::NAT*	In this study
TBL513	*MAT***a** *cdh1Δ:: NEO CDH1::NAT*	In this study
TBL575	*MATα cdh1Δ::NEO* P_{ACTIN}*-CDH1-Myc::Hyg*	In this study
TBL580	*MAT***a** *cdh1Δ::NEO* P_{ACTIN}*-CDH1-Myc::Hyg*	In this study
TBL630	*MATαcdh1Δ::NEO* P_{H3}*-GFP-Cdh1::NAT*	In this study

Perfect, J.R.; Ketabchi, N.; Cox, G.M.; Ingram, C.W.; Beiser, C.L. Karyotyping of *Cryptococcus neoformans* as an epidemiological tool. *J Clin Microbiol* **1993**, *31*, 3305-3309.

Nielsen, K.; Cox, G.M.; Wang, P.; Toffaletti, D.L.; Perfect, J.R.; Heitman, J. Sexual cycle of *Cryptococcus neoformans* var. grubii and virulence of congenic a and alpha isolates. *Infect Immun* **2003**, *71*, 4831-4841.

Table S2. Plasmids used in this study.

**Plasmids**	**Bacterial Marker**	**Description**
pJAF1	Amp^r Plasmid harboring *NEO* marker	Fraser et al., 2003
pCN19	Amp^r Plasmid harboring *GFP* under histone H3 promoter	Price et al., 2008
pTBL1	Amp^r Vector harboring *NAT* marker	Fan et al., 2019
pTBL153	Amp^r Vector for P_{Actin}*-GRP1*-HA-*NAT* for *GRP1* overexpression	Han et al., 2023
pTBL404	Amp^r Vector for P_{CDH1}*-CDH1-NAT* for complementary	In this study
pTBL405	Amp^r Vector for P_{ACTIN}*- Myc-Hyg* for complementary	In this study
pTBL435	Amp^r Vector for P_{ACTIN}*-GFP*-Cdh1-*NAT* for Cdh1 localization	In this study
pTBL443	Amp^r Vector for P_{ACTIN}-Cdh1-Myc for *CDH1* overexpression	In this study

Fraser, J.A.; Subaran, R.L.; Nichols, C.B.; Heitman, J. Recapitulation of the sexual cycle of the primary fungal pathogen Cryptococcus neoformans var. gattii: implications for an outbreak on Vancouver Island, Canada. *Eukaryot Cell* **2003**, *2*, 1036-1045.

Price, M.S.; Nichols, C.B.; Alspaugh, J.A. The Cryptococcus neoformans Rho-GDP dissociation inhibitor mediates intracellular survival and virulence. *Infect Immun* **2008**, *76*, 5729-5737, doi:10.1128/IAI.00896-08.

Fan, C.L.; Han, L.T.; Jiang, S.T.; Chang, A.N.; Zhou, Z.Y.; Liu, T.B. The Cys2His2 zinc finger protein Zfp1 regulates sexual reproduction and virulence in Cryptococcus neoformans. *Fungal Genet Biol* **2019**, *124*, 59-72, doi:10.1016/j.fgb.2019.01.002.

Han, L.; Wu, Y.; Xiong, S.; Liu, T. Ubiquitin Degradation of the AICAR Transformylase/IMP Cyclohydrolase Ade16 Regulates the Sexual Reproduction of Cryptococcus neoformans. *J Fungi (Basel)* **2023**, *9*, doi:10.3390/jof9070699.

Table S3. Primers used in this study.

Primers	Description	Sequence(5'-3')
TL17	M13 F	GTAAAACGACGGCCAG
TL18	M13 R	CAGGAAACAGCTATGAC
TL19	*NEO* split F	GGGCGCCCGGTTCTTTTTGTCA
TL20	*NEO* split R	TTGGTGGTCGAATGGGCAGGTAGC
TL217	*GAPDH* qRT-PCR F	TGAGAAGGACCCTGCCAACA
TL218	*GAPDH* qRT-PCR R	ACTCCGGCTTGTAGGCATCAA
TL1534	Mouse GAPDH qRT-PCR F	TGTGTCCGTCGTGGATCTGA
TL1535	Mouse GAPDH qRT-PCR R	TTGCTGTTGAAGTCGCAGGAG
TL1807	CXCL1 qRT-PCR F	GGGAGGCTGTGTTTGTATGT
TL1808	CXCL1 qRT-PCR R	CAGTCCTTTGAACGTCTCTGT
TL1809	CCL2 qRT-PCR F	GAAGGAATGGGTCCAGACATAC
TL1810	CCL2 qRT-PCR R	TCACACTGGTCACTCCTACA
TL1811	CXCL10 qRT-PCR F	ATGGCTGTCCTAGCTCTGTA
TL1812	CXCL10 qRT-PCR R	CTTGGGAAGATGGTGGTTAAGT
TL1813	CCL17 qRT-PCR F	AGGGATGCCATCGTGTTT
TL1814	CCL17 qRT-PCR R	AAAGGGAACAGGGACTTCTG
TL1815	CCL20 qRT-PCR F	CAGCCCAAGGAGGAAATGAT
TL1816	CCL20 qRT-PCR R	CCCATGGATTGTGGGAAATTAAC
TL1828	*CDH1* KO F1	GCAATCTGTGGTGAGGCAAAACTT
TL1829	*CDH1* KO R1	CTGGCCGTCGTTTTACGACGAGCGGATACGGCGAGAG
TL1830	*CDH1* KO F2	GTCATAGCTGTTTCCTGTTATGTCCTGATGTGCTGGCTACG
TL1831	*CDH1* KO R2	ACACACCTCGAACAATGAAAGTGAC
TL1832	*CDH1* KO F3	TTTACATCATCGCCCACCACCTAT
TL1833	*CDH1* KO R3	CTTATCTGCCGTACCACCTCCACT
TL1834	*CDH1* KO F4	AACGGGTGGGAAGAGACGAATAAT
TL1945	*CDH1*-Myc (Actin) F1	CGCCCAACATGTCTGGATCCATGTCCATTTACGATACAGAGT
TL1947	*CDH1* comp F	GATATCGAATTCCTGCAGCCCGGGGGATCCGCAATCTGTGGTGAGGCAAAACTT
TL1948	*CDH1* comp R	CGGTGGCGGCCGCTCTAGAACTAGTGGATCCACACACCTCGAACAATGAAAGTGAC
TL2119	GFP-*CDH1* F1	GACGAGCTGTACGGATCCATGTCCATTTACGATACAGAGTTC
TL2120	GFP-*CDH1* R1	CTGGCGGCCGTTACTAGTTCATCTCAATCCACCGAAGCCACC
TL2135	*CDH1* Q-PCR F1	AGGTGGTACGGCAGATAA
TL2136	*CDH1* Q-PCR R1	GACACGATAGTTGTGACCAG
TL2192	*CDH1*-Myc (Actin) R1	TAAGTTTTTGTTCGGATCCTCTCAATCCACCGAAGCCACCCA
TL2466	*APP1 Q-PCR F1*	TCGACTCGACCATGTGAATG
TL2467	*APP1 Q-PCR R1*	ATCAATGTTCGCAGCTCCT
